# Evaluations of Clinical Utilization of Metagenomic Next-Generation Sequencing in Adults With Fever of Unknown Origin

**DOI:** 10.3389/fcimb.2021.745156

**Published:** 2022-01-21

**Authors:** Zhang-fan Fu, Hao-cheng Zhang, Yi Zhang, Peng Cui, Yang Zhou, Hong-yu Wang, Ke Lin, Xian Zhou, Jing Wu, Hong-long Wu, Wen-hong Zhang, Jing-wen Ai

**Affiliations:** ^1^ Department of Infectious Diseases, Shanghai Key Laboratory of Infectious Diseases and Biosafety Emergency Response, National Medical Center for Infectious Diseases, Huashan Hospital, Fudan University, Shanghai, China; ^2^ BGI PathoGenesis Pharmaceutical Technology Co., Ltd., BGI-Shenzhen, Shenzhen, China; ^3^ BGI Wuhan Biotechnology, BGI-Shenzhen, Wuhan, China

**Keywords:** metagenomic next-generation sequencing, fever of unknown origin, precision diagnose, etiology, application

## Abstract

**Introduction:**

The diagnosis of infection-caused fever of unknown origin (FUO) is still challenging, making it difficult for physicians to provide an early effective therapy. Therefore, a novel pathogen detection platform is needed. Metagenomic next-generation sequencing (mNGS) provides an unbiased, comprehensive technique for the sequence-based identification of pathogenic microbes, but the study of the diagnostic values of mNGS in FUO is still limited.

**Methods:**

In a single-center retrospective cohort study, 175 FUO patients were enrolled, and clinical data were recorded and analyzed to compare mNGS with culture or traditional methods including as smears, serological tests, and nucleic acid amplification testing (NAAT) (traditional PCR, Xpert MTB/RIF, and Xpert MTB/RIF Ultra).

**Results:**

The blood mNGS could increase the overall rate of new organisms detected in infection-caused FUO by roughly 22.9% and 19.79% in comparison to culture (22/96 vs. 0/96; OR, ∞; p = 0.000) and conventional methods (19/96 vs. 3/96; OR, 6.333; p = 0.001), respectively. Bloodstream infection was among the largest group of those identified, and the blood mNGS could have a 38% improvement in the diagnosis rate compared to culture (19/50 vs. 0/50; OR, ∞; p = 0.000) and 32.0% compared to conventional methods (16/50 vs. 3/50; OR, 5.333; p = 0.004). Among the non-blood samples in infection-caused FUO, we observed that the overall diagnostic performance of mNGS in infectious disease was better than that of conventional methods by 20% (9/45 vs. 2/45; OR, 4.5; p = 0.065), and expectedly, the use of non-blood mNGS in non-bloodstream infection increased the diagnostic rate by 26.2% (8/32 vs. 0/32; OR, ∞; p = 0.008). According to 175 patients’ clinical decision-making, we found that the use of blood mNGS as the first-line investigation could effectively increase 10.9% of diagnosis rate of FUO compared to culture, and the strategy that the mNGS of suspected parts as the second-line test could further benefit infectious patients, improving the diagnosis rate of concurrent infection by 66.7% and 12.5% in non-bloodstream infection, respectively.

**Conclusion:**

The application of mNGS in the FUO had significantly higher diagnostic efficacy than culture or other conventional methods. In infection-caused FUO patients, application of blood mNGS as the first-line investigation and identification of samples from suspected infection sites as the second-line test could enhance the overall FUO diagnosis rate and serve as a promising optimized diagnostic protocol in the future.

## Introduction

Since 1961, Petersdorf has defined the fever of unknown origin (FUO) as a fever of 38.3°C or higher for 3 weeks or more on multiple occasions; the etiology cannot be determined despite appropriate investigation even after at least a week in the hospital (Petersdorf and Beeson, 1961). Over 200 disorders have been found in the differential diagnosis of FUO, which was divided into infection, non-infectious inflammatory disease (NIID), cancer, other conditions, and unresolved diagnosis (Mourad et al., 2003; Kucukardali et al., 2008; Horowitz, 2013). Due to the complicated clinical characteristics and absence of laboratory analysis indicators, the disease is difficult to be diagnosed and contributes to a tremendous cost on average of 40,295 US dollars during hospitalization (Szymanski et al., 2020). Because infection is one of the main causes of FUO (Fusco et al., 2019; Naito et al., 2019; Wright and Auwaerter, 2020; Wright et al., 2021), an optimized screening and diagnostic work flow for detecting pathogens of infection-caused FUO is needed.

Since the launch of the second-generation sequencing platform in early 2000, next-generation sequencing (NGS) technology has advanced at a rapid pace in the past 20 years. Nowadays, NGS may be performed for as low as $1,000 per sample (10.5 MB) to an average depth of 400× coverage (Yohe and Thyagarajan, 2017). Metagenomic next-generation sequencing (mNGS) is an inspection method that uses a next-generation sequencing platform to detect all genomes of environmental or patient samples (Chiu and Miller, 2019). Because of its unbiased and high throughput, mNGS has been used to study human microbial flora (Lagier et al., 2018), human pathogens (Gu et al., 2019), environmental microflora (ocean or soil) (Zhao & Bajic, 2015; Fierer, 2017), forensic field (Hampton-Marcell et al., 2017), etc. In addition, recent studies have reported their successful applications of mNGS in a variety of infectious disorders including central nervous system infection (Wilson et al., 2018; Wilson et al., 2019; Zhang et al., 2020), respiratory tract infection (Langelier et al., 2018), focal infection (Zhang et al., 2019), bloodstream infection (Gosiewski et al., 2017), etc. However, research directly evaluating the ability of mNGS in FUO is still limited, although previous study has exhibited its ability in increasing cost efficacy of FUO (Chai et al., 2018). Consequently, in this study, we undertook a retrospective cohort analysis on the overall clinical values of mNGS in the application of FUO patients in the east of China.

## Materials and Methods

### Study Design and Participants

**Figure 1:**
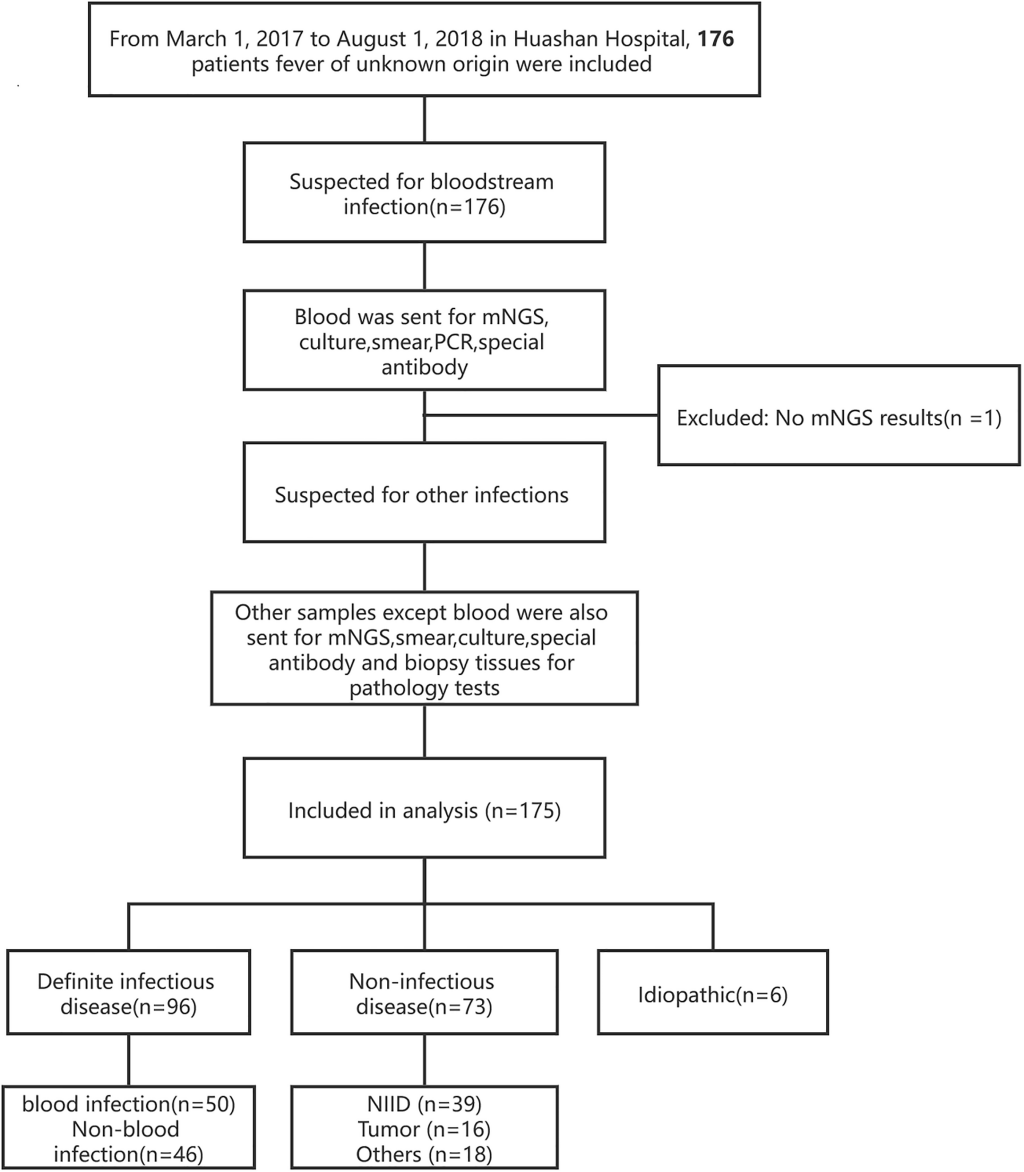
Overview of patients’ enrollment workflow. Flowchart of study.

This was a single-center, retrospective cohort study, in which all patients at the Huashan Hospital with the diagnosis of fever of unknown origin from March 7, 2017, to August 1, 2018 were enrolled (**Figure 1**). The final diagnosis of the 175 cases (**Table 1**) were evaluated by the experts group including three experienced physicians. Blood samples were collected after getting the consent of the patients or their surrogates and sent for mNGS and culture synchronously. Other examinations such as blood routine, blood biochemical tests, autoimmune antibody, and conventional pathogenic methods (including blood smear, latex agglutination test, serologic tests, and nucleic acid amplification testing (NAAT) (traditional PCR, Xpert MTB/RIF, and Xpert MTB/RIF Ultra) were performed according to the clinical necessity. If the patients were suspected of having infections other than bloodstream infections, specimens of suspected infected sites will also be sent for mNGS and the above-mentioned methods.

**Table 1:** Distribution of 175 FUO cases.

Disease	No.	Disease	No.	Disease	No.	Disease	No.	Disease	No.	Disease	No.
Infection	96*			NIID	39	Tumor	16	Others	18	Idiopathic	6
Bloodstream infections	50	Nasosinusitis	1	Vasculitis	14	Lymphoma	10	Allergic reaction	3		
Lower respiratory infections	17	Severe hepatitis	1	UCTD	6	Thyroid cancer	2	Drug fever	3		
Urinary infections	6	Tuberculous pleurisy	1	Adult-onset Still’s disease	6	Leukemia	1	Autoimmune encephalitis	2		
Central nervous system infections	6	Tuberculosis	1	Reactive arthritis	3	Bladder cancer	1	Fatigue syndrome	2		
Biliary duct infections	3	Vertebra infection	1	CTD	3	Bone metastatic cancer	1	Post-infection allergy	2		
Skin and soft tissue infections	3			SLE	2	HLH	1	Autoimmune hepatitis	1		
Endocarditis	3			polymyalgia rheumatica	2			Crohn disease	1		
Abdominal infections	1			Interstitial lung disease	1			Dysfunction of autonomic nerve	1		
Impetigo	1			Rheumatoid arthritis	1			Seizures	1		
Lymphangitis	1			Schnitzler syndrome	1			Systemic inflammatory response syndrome	1		
Lymphnoditis	1							Hashimoto thyroiditis	1		

*Some patients with infectious diseases have more than two infections, so the total number of cases of all infectious diseases exceeds 96.

UCTD, undefined connective tissue disease; CTD, connective tissue disease; SLE, systemic lupus erythematosus; HLH, hemophagocytic lymph histiocytosis.

### Criteria of Positive Detection Criteria for mNGS Test

Each sample’s sequencing results were divided into two tables, one for bacteria/fungi and the other for viruses. The specifically mapped read number (SMRN) of each microbial taxonomy was standardized to SMRN per 20 million (M) of total sequencing reads (SDSMRN, standardized SMRN).


SDSMRN=SMRN×20 million/total reads.

A bacterial/fungal was considered positively detected if (i) it was among the top 10 genera with the highest SDSMRN; (ii) within its genus, it was ranked top; (iii) its SDSMRN >1; and (iv) it was a commonly reported bloodstream infection pathogen.

A virus was judged positively detected if (i) if a virus belonged to the top 3 with the highest SDSMRN and (ii) it had a SDSMRN >5.

Because of the low yield of DNA extraction and relatively limited risk of contamination, pathogens such as *Mycobacterium* spp., *Nocardia* spp., *Brucella* spp., and others were considered identified if (i) it was one of the top 20 genera with the highest SDSMRN, (ii) it positioned first within its genus, and (iii) it had a SDSMRN >1. For the detection of pathogens within the Enterobacteriaceae family, only the species with highest SDSMRN was considered as a positive detection.

The clinical interpretation of sequencing results was mainly based on the symptoms, physical signs, and uses of drugs. The final diagnosis of the 175 cases was evaluated by the expert’s group including three experienced physicians who worked alone.

### Criteria of Fever of Unknown Origin

- Classic fever of unknown origin: a group of diseases that cannot be diagnosed after 1 week of comprehensive physical examination in the outpatient or hospitalization. Fever lasts for more than 3 weeks, and oral temperature is >38.3°C at least three times (or at least three times the temperature fluctuates >1.2°C within 1 day);
- Inpatients of fever of unknown origin: The patient had no fever when admitted but had a fever for more than 3 days after admission. The oral temperature was >38.3°C at least three times (or body temperature fluctuates >1.2°C at least three times within 1 day);
- Neutropenic patients with fever of unknown origin: The patient has agranulocytosis (neutrophil count <0.5 × 10^9^ cells/L); fever that lasts more than 3 days, oral temperature >38.3°C (or body temperature fluctuates >1.2°C, within 1 day); the body fluid specimens were cultured for >48 h, and the results were negative.

### Metagenomic Next-Generation Sequencing and Data Analysis

#### Sample Processing and Nucleic Acid Extraction

Three to five milliliters of the blood sample was drawn in an ethylenediaminetetraacetic acid (EDTA) anticoagulant tube (BD Vacutainer^®^ EDTA tubes) at room temperature within 12 h before plasma separation and centrifuged at 400*g* for 20–30 min at 20°C. The plasma from the patients was transferred to two new sterile 1.8-ml tubes and kept at −80°C until DNA extraction. Other samples of latent infected parts, including sputum, bronchoalveolar lavage fluid (BALF), cerebrospinal fluid (CSF), urine, puncture fluid, pleural fluid, NPS, and tissues (lung tissues, bone marrow, lymph nodes, and aortic valve vegetations) were collected according to standard procedures and also stored at −80°C. Before extraction, sputum specimens were liquefied and NPS was submerged in 3 ml preservation solution, while other samples went straight to the next operation.

Each sample was used for DNA extraction. For all liquid samples except plasma, 600 μl was transferred into a fresh 1.5 ml microcentrifuge and mixed with 1 g 0.5 mm BioSpec beads (0.5 mm dia. zirconia/silica, Cat. No. 11079105z) before being agitated vigorously at 2,800–3,200 rpm for 30 min on a horizontal platform on a Vortex-Genie 2 Vortex Mixer 12 (Scientific Industries, USA). Cell-free DNA was extracted directly from plasma without cell-wall-breaking treatment. Total DNA was extracted with TIANamp Micro DNA Kit (DP316, Tiangen Biotech, Beijing, China).

Samples from patients suspected with viral infection at the time of sample collection went through RNA extraction. Total RNA was extracted with QIAamp Viral RNA Mini Kit (52906, Qiagen, China) according to the manufacturer’s instructions. With the SuperScript II Reverse Transcription Kit (18064-014, Invitrogen, China), the RNA was reverse transcribed and synthesized into double-stranded complementary DNA (ds cDNA).

DNA libraries were constructed *via* DNA fragmentation, end repair, A-tailing addition, adapter ligation, and PCR amplification. The Agilent 2100 and Qubit 2.0 system was used to performed quality control (200–300 bp, >2 ng/μl), and qualified libraries were sequenced on BGISEQ-2000 platform with single-end 50 bp strategy. At least 20 million reads were produced from each sample. To control the sequencing quality and contamination of each sequencing run, we added positive and negative control (HeLa cell lines with or without *Acinetobacter baumannii*) in each run.

#### Bioinformation Pipeline

High-quality sequencing data were generated by removing low-quality and short (length <35 bp) reads. Next, the clean reads after quality filtering were mapped to a human reference database including hg19 and Yanhuang genome sequence using Burrows–Wheeler alignment (Version 0.7.10). Remaining reads were aligned to the non-redundant bacterial, virus, fungal, and parasite databases using Burrows–Wheeler alignment (Version 0.7.10). The mapped data were processed for advanced data analysis. The genome databases were downloaded from National Center for Biotechnology Information (NCBI) (ftp://ftp.ncbi.nlm.nih.gov/genomes/). RefSeq contains 4,061 whole genome sequence of viral taxa, 2,473 bacterial genomes or scaffolds, 199 fungi related to human infection, and 135 parasites associated with human diseases. We uploaded the raw data onto China National GeneBank (CNP0000607).

### Statistical Analysis

In this article, the pathogens extra detection rate (PEDR) of mNGS and the improved diagnostic rate (IDR) of mNGS were calculated by the following methods:

Pathogens extra detection rate:


$$PEDR_{mNGS/culture}=\frac{true\;mNGS\;positive\;cases-culture\;positive\;cases}{clinical\;definate\;infectious\;cases};$$

$$PEDR_{mNGS/conventional\;methods}=\frac{true\;mNGS\;positive\;cases-culture\;positive\;cases}{clinical\;definate\;infectious\;cases}$$

where the true mNGS-positive cases were those in consistent with clinical judgement.

Improved diagnostic rate:


$$IDR_{bloodstream\;infection}=IDR_{BSI}=\frac{true\;mNGS\;positive\;cases\;BSI-culture\;positive\;cases\;BSI}{suspected\;BSI\;cases};$$

$$IDR_{cocurrent\;infection\;with\;bloodstream\;\inf ection}=\;IDR_{co-BSI}=\frac{true\;mNGS\;positive\;cases\;of\;co-BSI-culture\;positive\;cases\;of\;CO-BSI}{suspected\;co-BSI};$$

$$IDR_{non-bloodstream\;infection}=\;IDR_{non-BSI}=\frac{true\;mNGS\;positive\;cases\;of\;non-BSI-culture\;positive\;cases\;of\;non-BSI}{suspected\;non-BSI}$$

The continuous variants of baseline characteristics were analyzed using the Student’s t-test with their odds ratios (ORs) and 95% confidence intervals (95% CIs). We analyzed McNemar test and kappa test to compare differences across subgroups. Statistical analyses and figures were conducted using the SPSS statistical package 12.0 software, Excel 2019, Xmind 8.0 version, and R 4.1.0 version.

## Results

### General Characteristics of Enrolled Patients

A total of 176 patients with the diagnosis of FUO were enrolled in the study and underwent clinically baseline screening in Huashan Hospital affiliated to Fudan University from March 7, 2017 to August 1, 2018. We excluded one case for low quantity of nucleic acid verification of mNGS and finally enrolled 175 individuals. According to the 175 patients’ final diagnosis, 96 of these 175 patients were classified to the group of infectious disease (ID). Seventy-three patients were categorized into the non-infectious disease (NID), and the other six patients were idiopathic group (IG) (**Figure 1**). Additionally, the NID group was further classified to NIID (n = 39), tumor (n = 16), and other diseases (n = 18). All the baseline characteristics of 175 patients are shown in **Table 2**, and there was not any significant difference in baseline data including gender, age, count of white blood cell (WBC), C-reactive protein (CRP), erythrocyte sedimentation rate (ESR), and procalcitonin (PST) between ID and NID groups.

**Table 2:** The baseline of enrolled patients.

	Overall	Infection	Non-infection	p-values
**N**	169	96	73	
**Age [mean (SD)]**	47.87 (16.35)	47.33 (15.43)	48.58 (17.57)	0.626
**Gender = Male (%)**	103 (60.9)	62 (64.6)	41 (56.2)	0.341
**WBC [mean (SD)]**	27.98 (140.52)	23.64 (127.94)	34.25 (157.79)	0.641
**PLT [mean (SD)]**	232.96 (121.58)	241.44 (126.61)	221.03 (114.05)	0.306
**Neutrophil [mean (SD)]**	5.78 (3.63)	6.14 (3.94)	5.26 (3.08)	0.149
**ESR [mean (SD)]**	32.13 (23.44)	32.37 (25.00)	31.77 (21.09)	0.877
**CRP [mean (SD)]**	53.46 (51.94)	56.69 (53.63)	48.59 (49.32)	0.347
**Ferritin [mean (SD)]**	894.32 (728.53)	910.26 (746.13)	870.84 (707.96)	0.758
**PCT [mean (SD)]**	1.60 (9.19)	1.67 (8.66)	1.49 (10.00)	0.909
**RBC [mean (SD)]**	24.35 (198.97)	34.85 (258.34)	9.17 (19.01)	0.426

*The remain six are idiopathic; they do not belong to infection or non-infection.

### Distribution of Identified Pathogens by mNGS

**Figure 2:**
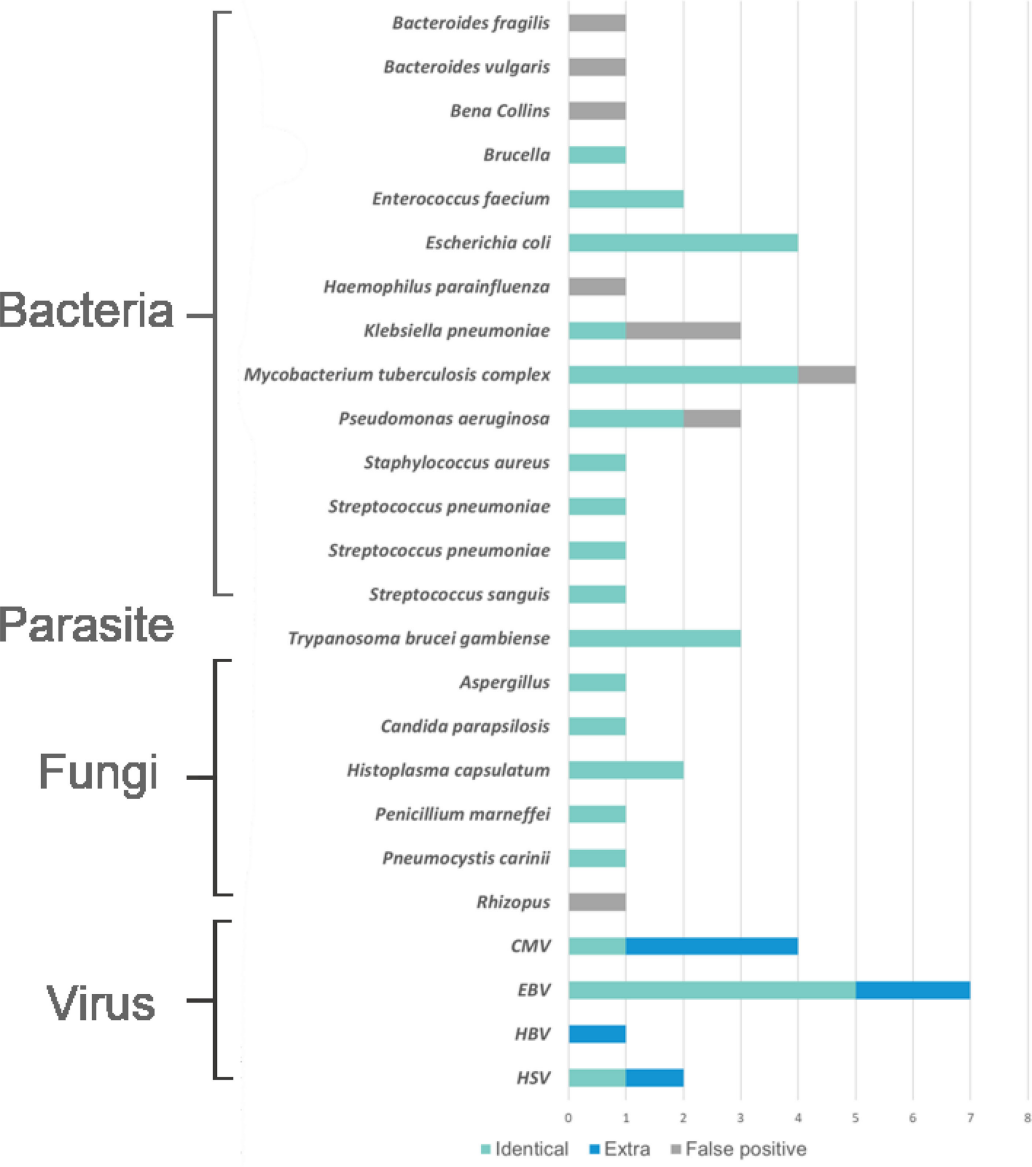
The distribution of pathogens detected by metagenomic next generation sequencing (mNGS). The distributions were divided into four parts, namely, bacteria, parasites, fungi, and viruses, and each part consisted of identical detection (the blue), extra detection (the green), and false positive detection (the gray).

**Figure 3:**
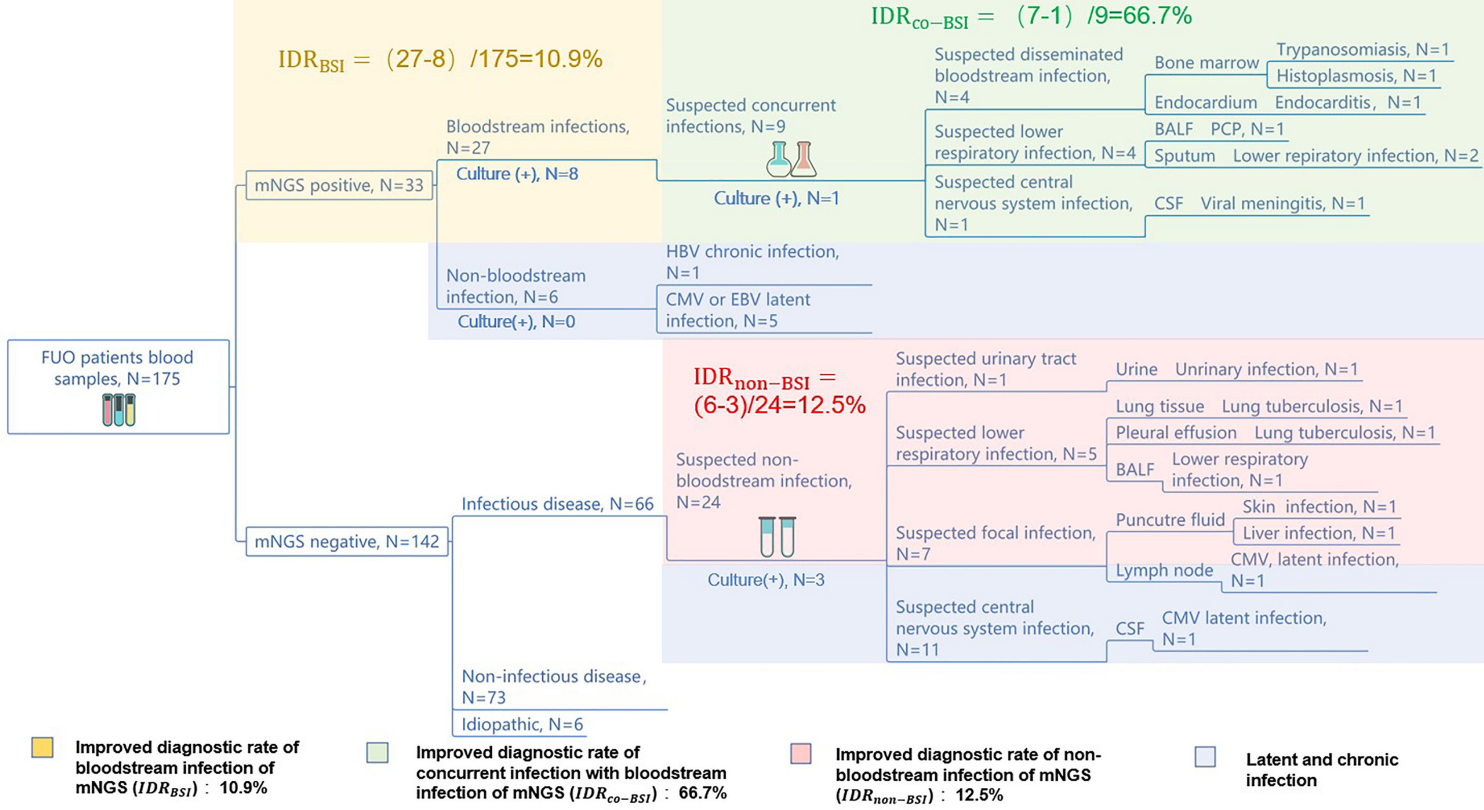
mNGS improves the diagnosis rate of infectious diseases. The diagnostic workflows of the 175 FUO patients’ pathogen identification. The yellow, green, red, and purple part show the mNGS’ improving diagnostic rate of bloodstream infections, concurrent infection with bloodstream infection, non-bloodstream infection, and latent or chronic infection, respectively. The numbers of improved rate of mNGS were calculated by: $IDR=\frac{true\;mNGS\;positive\;cases-culture\;positive\;cases}{suspected\;infectious\;cases},$ .

In general, the results of pathogens detected by mNGS showed that the virus was the most prevalent pathogens, in which the most frequently detected was *EBV* and then *CMV* (**Figure 2**). The top identified bacteria were *Mycobacterium tuberculosis* complex and *Escherichia coli*, and we also detected a wide range of fungi, such as *Penicillium marneffei*, *Aspergillus spp*., etc. mNGS was proved to have advantage over culture mainly in the identification of causal agents that cannot be cultured or had time-consuming culture process, such as *EBV*, *Aspergillus spp*., and *MTB* complex. However, mNGS had some more false-positive cases than that in culture in our study (**Supplementary Tables 1A**–**C**). **Figure 3**

### Diagnostic Performance on Pathogens Extra Detection Rate of mNGS, Compared to Culture and Other Conventional Methods in Using Blood Samples in Infection-Caused FUO

In order to evaluate the diagnostic performance of mNGS, we calculated a set of PEDR_mNGS/culture_. The overall PEDR_mNGS/culture_ was 22.9%, PEDR_mNGS/culture_ of bloodstream infection was 38%, but PEDR_mNGS/culture_ of non-bloodstream infection was only 6.5%. We concluded that the mNGS of the FUO patients’ blood samples might increase the pathogens extra detection rate of infectious diseases compared to that of culture alone by 22.9% (22/96 vs. 0/96; OR, ∞; p = 0.000) (**Table 3**). Bloodstream infection comprised the largest component of all the diseases, and the blood mNGS test could have a 38% improvement of pathogens extra detection rate compared to culture in the bloodstream infection (19/50 vs. 0/50; OR, ∞; p = 0.000) (**Table 3**), but the pathogens extra detection rate of blood mNGS test in non-bloodstream infection was limited only by 6.5% (**Table 3**).

**Table 3:** The diagnostic value of mNGS compared to culture in blood samples in FUO.

Disease	Samples	NGS(+)/culture(+)	NGS(+)/culture(−)	NGS(−)/culture(+)	NGS(−)/culture(−)	PEDR_mNGS/culture_ (%)
**Infectious disease**	96	8	22	0	66	22.9%
Blood infection	50	8	19	0	23	38.0%
Non-blood infection	46	0	3*	0	43	6.5%
**NIID**	39	0	1*	0	38	
**Tumor**	16	0	2*	0	14	
**Others**	18	0	0	0	18	
**Idiopathic**	6	0	0	0	6	

*These specimens additionally detect viruses such as CMV, EBV, and HBV, and virus chronic infection or in a latent infection is considered.

We further analyzed the consistency between mNGS and culture of blood samples. In both mNGS and culture, 8 of 96 cases tested positive for infectious disease, and 66 of 96 cases were negative. Twenty-two cases were mNGS test positive and culture negative. None of the samples had only culture-positive results (**Table 3**). In our analysis, the concordance of mNGS and culture was not so consistent (Kappa value = 0.342, p = 0.000; McNemar, p = 0.000), and the possible reason may be that the total diagnostic efficacy of mNGS is higher than that of culture by 22.9% (**Table 3**).

We also evaluated pathogens extra detection rate of mNGS compared to other conventional methods in blood samples (**Table 4**). The total PEDR_mNGS/conventional methods_ was 19.79%, the bloodstream infection of PEDR_mNGS/conventional methods_ was 32%, and the non-bloodstream infection’s PEDR_mNGS/conventional methods_ was 6.52%. Overall, the total pathogens extra detection rate of mNGS was higher than that of conventional methods (19/96 vs. 3/96; OR, 6.333; p = 0.001) by 19.79% in all diseases, particularly in bloodstream infection by 32.0% (16/50 vs. 3/50; OR, 5.333; p = 0.004), while the pathogens extra detection rate of blood mNGS in non-bloodstream infection showed limited efficacy by 6.52% (**Table 4**). mNGS was also outstanding in the exclusion of infectious disease than conventional methods, according to the negative results of the tests in the NID (**Tables 3**, **4**).

**Table 4:** The diagnostic value of mNGS compared to conventional methods in blood samples in FUO.

Disease	Samples	NGS(+)/CM(+)	NGS(+)/CM(−)	NGS(−)/CM(+)	NGS(−)/CM(−)	PEDR_mNGS/conventional methods_ (%)
**Infectious disease**	96	11	19	3	63	19.79%
Blood infection	50	11	16	3	20	32.0%
Non-blood infection	46	0	3*	0	43	6.52%
**NIID**	39	0	1*	0	38	
**Tumor**	16	0	2*	0	14	
**Others**	18	0	0	0	18	
**Idiopathic**	6	0	0	0	6	

*These specimens additionally detect viruses such as CMV, EBV, and HBV, and virus chronic infection or in a latent infection is considered.

### Diagnostic Performance on Pathogens Extra Detection Rate of mNGS, Compared to Conventional Methods in Using Non-Blood Samples in Infection-Caused FUO

We also obtained a set of PEDR_mNGS/conventional methods_ of non-blood samples to evaluate the diagnostic efficacy of mNGS. The total PEDR_mNGS/conventional methods_ of non-blood samples in all infections was 20%, and non-bloodstream infection’s PEDR_mNGS/conventional methods_ was 26.2%, while the bloodstream infection’s PEDR_mNGS/conventional methods_ was only 7.7%, respectively. The use of mNGS in non-blood samples showed better performance than conventional methods in infectious diseases (9/45 vs. 2/45; OR, 4.5; p = 0.065) and improved the pathogens extra detection rate by 20%. What is more, especially in non-bloodstream infection, the application of non-blood samples mNGS increased the pathogens extra detection rate by 26.2% compared to conventional methods (8/32 vs. 0/32; OR, ∞; p = 0.008) (**Table 5**).

**Table 5:** The diagnostic value of mNGS compared to conventional methods in non-blood samples in FUO.

Disease	Samples	Sample type	NGS(+)/CM(+)	NGS(+)/CM(−)	NGS(−)/CM(+)	NGS(−)/CM(−)	PEDR_conventional methods_ (%)
**Infection**	45	/	8	9	2	26	20%
bloodstream infection	13	/	0	1	1	11	7.70%
	4	CSF	0	0	0	4	0.00%
	4	Tissue	0	1	1	2	25.00%
	3	Puncture fluid	0	0	0	3	0.00%
	1	Pleural fluid	0	0	0	1	0.00%
	1	BALF	0	0	0	1	0.00%
Non-blood stream infection	32	/	8	8	0	16	26.20%
	12	Tissue	2	3	0	7	25.00%
	9	CSF	1	2	0	6	22.22%
	2	Puncture fluid	2	0	0	0	0.00%
	4	Sputum	1	1	0	2	25.00%
	3	BALF	2	0	0	1	0.00%
	1	Urine	0	1	0	0	100.00%
	1	Pleural fluid	0	1	0	0	100.00%

### The Application of mNGS in Assisting FUO Clinical Decision-Making and Improving Diagnostic Rate

To assist the clinical decision-making, we have summarized a diagnostic work flow (**Supplementary Figure 1**) of the 175 FUO patients comparing mNGS with culture. We have found that the application of blood mNGS as the first-line examination is an effective strategy in the diagnosis of infection-caused FUO, including bloodstream infection, concurrent infection with bloodstream infection, and non-bloodstream infection. We reported an IDR_BSI_ of 10.9%, an IDR_CO-BSI_ of 66.7%, and an IDR_non-BSI_ of 12.5%, respectively. Compared to culture, blood mNGS test can improve the diagnostic rate of bloodstream infection by 10.9%. The application of mNGS as the second-line test in the suspected infectious part can also improve the diagnostic rate of the FUO patients by 66.7% among those who have concurrent infection with BSI. Moreover, mNGS can diagnose more non-bloodstream infectious diseases than culture by 12.5%.

Furthermore, mNGS may diagnose the cases of latent or chronic viral infection (HBV, CMV, EBV, etc.), and those pathogens may be not the main cause of FUO but assist the later clinical decision-making.

## Discussion

The global burden of febrile illness is still difficult to quantify. Currently, the cause of fever disease is still not identified in approximately 9–51% of patients (Bleeker-Rovers et al., 2007; Shi et al., 2013; Kabapy et al., 2016). Despite advances in diagnostic methods, the proportion of patients with undiagnosed FUO after the systematic examination has not decreased. The reason may be that with the improvements of medical care and diagnostic tools, diagnosis rates of common diseases are improved. However, cases that meet the classic definition of FUO are becoming increasingly complex (Shi et al., 2013; Robine et al., 2014).

Although the proportion of different causative diseases of FUO has changed over time, infectious factors remain the primary cause (Fusco et al., 2019). The complex infectious diseases pose a challenge for diagnostic and therapeutic determination. Unbiased mNGS represents a powerful tool to fill the gaps in our understanding of the etiology of FUO, thus informing the improvement of diagnostic algorithms. The previous study mentioned above has proved that mNGS can provide a 30%–50% extra detection rate in multiple types of infection such as focal infections, central nervous infections, and respiratory infections.

In this study, we conducted a retrospective cohort study to evaluate the diagnostic value of mNGS techniques in patients who were diagnosed with FUO. The PEDRmNGS/conventional methods of blood mNGS in infectious disease is 19.79%, which is higher than that of conventional methods, particularly in BSI that is 32.0%. Also, the PEDRconventional methods of non-blood mNGS in infection is 20%, which is also higher than that of conventional methods, especially in non-BSI that is 26.2%. Thus, the application of mNGS can help improve the clinical diagnosis by 38% in bloodstream infection and 6.5% in non-bloodstream infection. Our study showed that mNGS may act as a promising tool for the diagnosis of FUO patients. However, the results implied that if only blood samples are tested in patients of FUO, the clinical value of mNGS is relatively low. Only by collecting effective specimens from the part suspected of specific infection can the detection rate of the etiology be improved. The assessment and identification of etiology have improved the medical management of patients with FUO, helping to avoid the administration of unnecessary antibiotic therapy (Wylie et al., 2012).

What is more, mNGS is considered to have the latent to be used as an all-in-one diagnostic test, helping differentiate infections and non-infections, identify causative agents, and discover novel or emerging pathogens (Jerome et al., 2019). However, the expensive cost made mNGS not act as an all-purpose tool to resolve any uncertainty, but rather as a technology that cloud be used in specific contexts, such as seriously ill cases, or as a supplement to some special clinical cases (Chai et al., 2018).

In our study, we found the problem of the low detection rate of RNA virus, intracellular bacteria, and fungi. We listed the probable reasons of the low detection rate of RNA virus as follows. We apply the RNA meta-transcriptomics test process in all respiratory samples and did not apply the process in other samples considering the high expense of the mNGS process and higher RNA virus detection rate in respiratory samples. In addition, since we did not remove the human cellular RNA before the mNGS sequencing process, the virus’ RNA genome is difficult to be detected compared to the human’s cellular RNA yield. Thus, the detection rate of the RNA virus is relatively low in our study. In order to get a higher detection rate of RNA viruses and make a more accurate diagnosis of FUO, we need to perform the RNA meta-transcriptomics procedure to the samples and reassess the diagnostic rate in the further study.

The low detection rate of the fungi was mainly caused by the rigid cell walls; thus, many studies recommended adding bead beating to solve the problem. But at the same time, it also increases the host background of human DNA (Gu et al., 2021). Moreover, some studies are trying to add lyticase to increase the fungi’s DNA release.

For the intracellular bacteria, the low detection rate is related to the limited copies of intracellular bacteria in plasma. Some studies have improved the protocol of the whole process by increasing the DNA and RNA’s release of samples by resuspension in lysis buffer [10 mM Tris–HCl, 25 mM EDTA, 1% sodium dodecyl sulfate (SDS), 0.8 mg/ml, proteinase K] with a mixture of 1.0 mm zirconia and 0.1 mm of silica/zirconia beads, then going on the instructions of DNA extraction (Batinga et al., 2017). On the other hand, deepening the sequencing coverage of samples can be another method (Zhou et al., 2019).

Our study still had some limitations. First, this is a retrospective study with a small sample size in one single center; for the limited diseases and the patients’ number, a larger cohort from some multicenter study may be needed to further confirm our results. Second, there are still six patients who have not finally been diagnosed in our cohort, which implied that the mNGS alone cannot act as the final diagnostic tool for the FUO diseases. To get the final outcome of those patients, we may need follow-up information in a longer period.

In conclusion, this single-center retrospective study found that using mNGS to detect pathogens in FUO holds higher diagnostic sensitivity than culture or other conventional methods. In FUO patients, particularly those suspected of infectious illnesses, utilizing blood mNGS as the first-line investigation can increase the diagnosis rate; using samples from other suspected infection sites as the optional second-line test could further improve diagnostic ability. However, because mNGS technology is expensive and the FUO disease spectrum is relatively broad, including a large number of non-infectious diseases, accurate and effective selection of target samples and specimens could contribute to better efficacy use of mNGS’ unbiased and precise characteristics and is the key for significantly improving the patients’ diagnostic rate and success rate of treatment. Overall, the application of mNGS in FUO patients serve as a promising optimized diagnostic protocol in the future.

## Data Availability Statement

The original contributions presented in the study are included in the article/**Supplementary Material**. Further inquiries can be directed to the corresponding author.

## Ethics Statement

The study was approved by the Huashan Hospital ethical committee the methods were carried out in accordance with the approved guidelines of the institution. The ethical number is No. Ky2017-338.

## Author Contributions

JA conceived and designed the study. ZF and HZ analyzed the data and collected the related clinical information. HWa and KL conducted clinical work associated with the study. YZhou, PC, and HWu provided technical support. YZhang and XZ wrote the draft. JA and WZ made a final revision of the manuscript for important intellectual content. All authors contributed to the article and approved the submitted version.

## Funding

This work was supported by the National Natural Science Foundation of China (82002141), Shanghai Youth Science and Technology Talents Sailing Project (20YF1404300) and Special Projects for Medical Innovation Research (21Y11902100).

## Conflict of Interest

Authors YZ and HW were employed by company BGI-Shenzhen.

The remaining authors declare that the research was conducted in the absence of any commercial or financial relationships that could be construed as a potential conflict of interest.

## Publisher’s Note

All claims expressed in this article are solely those of the authors and do not necessarily represent those of their affiliated organizations, or those of the publisher, the editors and the reviewers. Any product that may be evaluated in this article, or claim that may be made by its manufacturer, is not guaranteed or endorsed by the publisher.

## References

[B1] Batinga M. C. A. Dos Santos J. C. Lima J. T. R. Bigotto M. F. D. Muner K. Faita T.. (2017). Comparison of Three Methods for Recovery of Brucella Canis DNA From Canine Blood Samples. J. Microbiol. Methods 143, 26–31. doi: 10.1016/j.mimet.2017.08.019 28864247

[B2] Bleeker-Rovers C. P. Vos F. J. de Kleijn E. Mudde A. H. Dofferhoff T. S. M. Richter C.. (2007). A Prospective Multicenter Study on Fever of Unknown Origin: The Yield of a Structured Diagnostic Protocol. Medicine 86 (1), 26–38. doi: 10.1097/MD.0b013e31802fe858 17220753

[B3] Chai J. H. Lee C. K. Lee H. K. Wong N. Teo K. Tan C. S.. (2018). Cost-Benefit Analysis of Introducing Next-Generation Sequencing (Metagenomic) Pathogen Testing in the Setting of Pyrexia of Unknown Origin. PloS One 13 (4), e0194648. doi: 10.1371/journal.pone.0194648 29664913 PMC5903630

[B4] Chiu C. Y. Miller S. A. (2019). Clinical Metagenomics. Nat. Rev. Genet. 20 (6), 341–355. doi: 10.1038/s41576-019-0113-7 30918369 PMC6858796

[B5] Fierer N. (2017). Embracing the Unknown: Disentangling the Complexities of the Soil Microbiome. Nat. Rev. Microbiol. 15 (10), 579–590. doi: 10.1038/nrmicro.2017.87 28824177

[B6] Fusco F. M. Pisapia R. Nardiello S. Cicala S. D. Gaeta G. B. Brancaccio G. (2019). Fever of Unknown Origin (FUO): Which Are the Factors Influencing the Final Diagnosis? A 2005-2015 Systematic Review. BMC Infect. Dis. 19 (1), 653. doi: 10.1186/s12879-019-4285-8 31331269 PMC6647059

[B7] Gosiewski T. Ludwig-Galezowska A. H. Huminska K. Sroka-Oleksiak A. Radkowski P. Salamon D.. (2017). Comprehensive Detection and Identification of Bacterial DNA in the Blood of Patients With Sepsis and Healthy Volunteers Using Next-Generation Sequencing Method - the Observation of DNAemia. Eur. J. Clin. Microbiol. Infect. Dis. 36 (2), 329–336. doi: 10.1007/s10096-016-2805-7 27771780 PMC5253159

[B8] Gu W. Deng X. Lee M. Sucu Y. D. Arevalo S. Stryke D.. (2021). Rapid Pathogen Detection by Metagenomic Next-Generation Sequencing of Infected Body Fluids. Nat. Med. 27 (1), 115–124. doi: 10.1038/s41591-020-1105-z 33169017 PMC9020267

[B9] Gu W. Miller S. Chiu C. Y. (2019). Clinical Metagenomic Next-Generation Sequencing for Pathogen Detection. Annu. Rev. Pathol. 14, 319–338. doi: 10.1146/annurev-pathmechdis-012418-012751 30355154 PMC6345613

[B10] Hampton-Marcell J. T. Lopez J. V. Gilbert J. A. (2017). The Human Microbiome: An Emerging Tool in Forensics. Microb. Biotechnol. 10 (2), 228–230. doi: 10.1111/1751-7915.12699 28244273 PMC5328825

[B11] Horowitz H. W. (2013). Fever of Unknown Origin or Fever of Too Many Origins? N. Engl. J. Med. 368 (3), 197–199. doi: 10.1056/NEJMp1212725 23323894

[B12] Jerome H. Taylor C. Sreenu V. B. Klymenko T. Filipe A. D. S. Jackson C.. (2019). Metagenomic Next-Generation Sequencing Aids the Diagnosis of Viral Infections in Febrile Returning Travellers. J. Infect. 79 (4), 383–388. doi: 10.1016/j.jinf.2019.08.003 31398374 PMC6859916

[B13] Kabapy A. F. Kotkat A. M. Shatat H. Z. Abd El Wahab E. W. (2016). Clinico-Epidemiological Profile of Fever of Unknown Origin in an Egyptian Setting: A Hospital-Based Stud-2010). J. Infect. Dev. Ctries 10 (1), 30–42. doi: 10.3855/jidc.7198 26829535

[B14] Kucukardali Y. Oncul O. Cavuslu S. Danaci M. Calangu S. Erdem H.. (2008). The Spectrum of Diseases Causing Fever of Unknown Origin in Turkey: A Multicenter Study. Int. J. Infect. Dis. 12 (1), 71–79. doi: 10.1016/j.ijid.2007.04.013 17629532

[B15] Lagier J. C. Dubourg G. Million M. Cadoret F. Bilen M. Fenollar F.. (2018). Culturing the Human Microbiota and Culturomics. Nat. Rev. Microbiol 16, 540–550. doi: 10.1038/s41579-018-0041-0 29937540

[B16] Langelier C. Zinter M. S. Kalantar K. Yanik G. A. Christenson S. O’Donovan B.. (2018). Metagenomic Sequencing Detects Respiratory Pathogens in Hematopoietic Cellular Transplant Patients. Am. J. Respir. Crit. Care Med. 197 (4), 524–528. doi: 10.1164/rccm.201706-1097LE 28686513 PMC5821905

[B17] Mourad O. Palda V. Detsky A. S. (2003). A Comprehensive Evidence-Based Approach to Fever of Unknown Origin. Arch. Intern. Med. 163 (5), 545–551. doi: 10.1001/archinte.163.5.545 12622601

[B18] Naito T. Tanei M. Ikeda N. Ishii T. Suzuki T. Morita H.. (2019). Key Diagnostic Characteristics of Fever of Unknown Origin in Japanese Patients: A Prospective Multicentre Study. BMJ Open 9 (11), e032059. doi: 10.1136/bmjopen-2019-032059 PMC6886908 31748308

[B19] Petersdorf R. G. Beeson P. B. (1961). Fever of Unexplained Origin: Report on 100 Cases. Medicine (Baltimore) 40, 1–30. doi: 10.1097/00005792-196102000-00001 13734791

[B20] Robine A. Hot A. Maucort-Boulch D. Iwaz J. Broussolle C. Sève P. (2014). Fever of Unknown Origin in the 2000s: Evaluation of 103 Cases Over Eleven Years. Presse Med. 43 (9), e233–e240. doi: 10.1016/j.lpm.2014.02.026 24985921

[B21] Shi X. C. Liu X. Q. Zhou B. T. Zhang L. F. Ma X. J. Deng G. H.. (2013). Major Causes of Fever of Unknown Origin at Peking Union Medical College Hospital in the Past 26 Years. Chin. Med. J. (Engl.) 126 (5), 808–812. 23489781

[B22] Szymanski A. M. Clifford H. Ronis T. (2020). Fever of Unknown Origin: A Retrospective Review of Pediatric Patients From an Urban, Tertiary Care Center in Washington, DC. World J. Pediatr. 16 (2), 177–184. doi: 10.1007/s12519-019-00237-3 30888665

[B23] Wilson M. R. O’Donovan B. D. Gelfand J. M. Sample H. A. Chow F. C. Betjemann J. P.. (2018). Chronic Meningitis Investigated via Metagenomic Next-Generation Sequencing. JAMA Neurol. 75 (8), 947–955. doi: 10.1001/jamaneurol.2018.0463 29710329 PMC5933460

[B24] Wilson M. R. Sample H. A. Zorn K. C. Arevalo S. Yu G. Neuhaus J.. (2019). Clinical Metagenomic Sequencing for Diagnosis of Meningitis and Encephalitis. N. Engl. J. Med. 380 (24), 2327–2340. doi: 10.1056/NEJMoa1803396 31189036 PMC6764751

[B25] Wright W. F. Auwaerter P. G. (2020). Fever and Fever of Unknown Origin: Review, Recent Advances, and Lingering Dogma. Open Forum Infect. Dis. 7 (5), ofaa132–ofaa132. doi: 10.1093/ofid/ofaa132 32462043 PMC7237822

[B26] Wright W. F. Simner P. J. Carroll K. C. Auwaerter P. G. (2021). Progress Report: Next-Generation Sequencing (NGS), Multiplex Polymerase Chain Reaction (PCR), and Broad-Range Molecular Assays as Diagnostic Tools for Fever of Unknown Origin (FUO) Investigations in Adults. Clin. Infect. Dis. 2021, ciab155. doi: 10.1093/cid/ciab155 33606012

[B27] Wylie K. M. Mihindukulasuriya K. A. Sodergren E. Weinstock G. M. Storch G. A. (2012). Sequence Analysis of the Human Virome in Febrile and Afebrile Children. PloS One 7 (6), e27735. doi: 10.1371/journal.pone.0027735 22719819 PMC3374612

[B28] Yohe S. Thyagarajan B. (2017). Review of Clinical Next-Generation Sequencing. Arch. Pathol. Lab. Med. 141 (11), 1544–1557. doi: 10.5858/arpa.2016-0501-RA 28782984

[B29] Zhang H. C. Ai J. W. Cui P. Zhu Y. M. Hong-Long W. Li Y. J.. (2019). Incremental Value of Metagenomic Next Generation Sequencing for the Diagnosis of Suspected Focal Infection in Adults. J. Infect. 79 (5), 419–425. doi: 10.1016/j.jinf.2019.08.012 31442461

[B30] Zhang Y. Cui P. Zhang H. C. Wu H. L. Ye M. Z. Zhu Y. M.. (2020). Clinical Application and Evaluation of Metagenomic Next-Generation Sequencing in Suspected Adult Central Nervous System Infection. J. Transl. Med. 18 (1), 199. doi: 10.1186/s12967-020-02360-6 32404108 PMC7222471

[B31] Zhao F. Bajic V. B. (2015). The Value and Significance of Metagenomics of Marine Environments. Preface. Genomics Proteomics Bioinf. 13 (5), 271–274. doi: 10.1016/j.gpb.2015.10.002 PMC4678774 26607677

[B32] Zhou X. Wu H. Ruan Q. Jiang N. Chen X. Shen Y.. (2019). Clinical Evaluation of Diagnosis Efficacy of Active Mycobacterium Tuberculosis Complex Infection via Metagenomic Next-Generation Sequencing of Direct Clinical Samples. Front. Cell Infect. Microbiol. 9, 351. doi: 10.3389/fcimb.2019.00351 31681628 PMC6813183

