# Supplementary material for: Evaluations of Clinical Utilization of Metagenomic Next-Generation Sequencing in Adults With Fever of Unknown Origin
Source: Front Cell Infect Microbiol. 2022 Jan 21;11:745156. doi: 10.3389/fcimb.2021.745156 (PMC8813867; doi:10.3389/fcimb.2021.745156)
Supplement: Supplementary file 1 [file DataSheet_1.pdf]

SFig1
175 FUO case diagnosis path

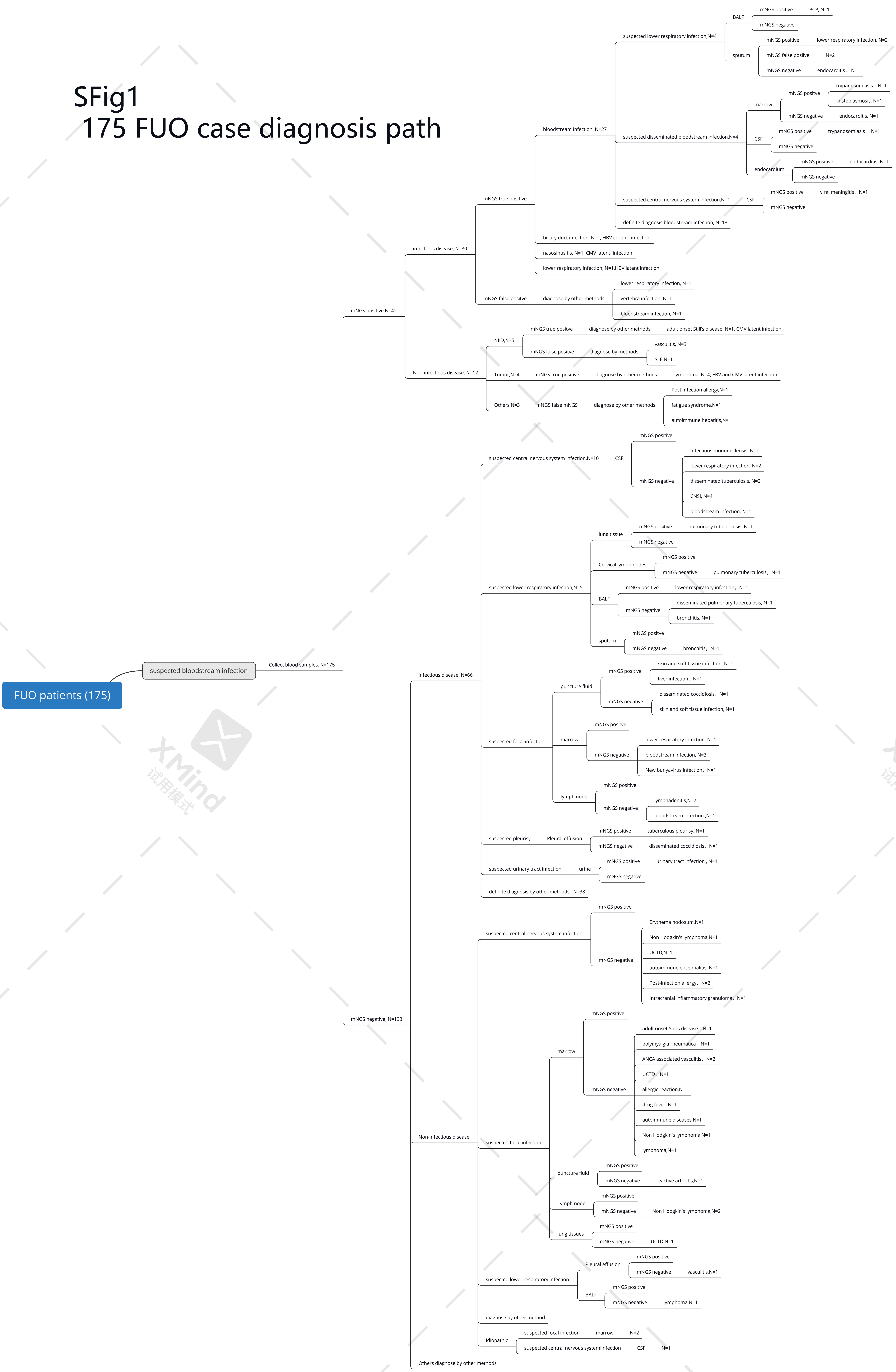

XMind 试用模式
