# Supplementary material for: Evaluations of Clinical Utilization of Metagenomic Next-Generation Sequencing in Adults With Fever of Unknown Origin
Source: Front Cell Infect Microbiol. 2022 Jan 21;11:745156. doi: 10.3389/fcimb.2021.745156 (PMC8813867; doi:10.3389/fcimb.2021.745156)
Supplement: Supplementary file 2 [file Table_1.docx]

**S1A. False positive cases in infection disease**

| **Case No** | **Final diagnosis** | **Pathogens detected by NGS** |
| --- | --- | --- |
| 7 | Pseudomonas aeruginosa blood infection | *Klebsiella pneumoniae* |
| 20 | CAEBV | *Mycobacterium Tuberculosis Complex* |
| 25 | Staphylococcus aureus blood infection | *Klebsiella pneumoniae*  *Pseudomonas aeruginosa* |
| 27 | Lower respiratory infection | *Mycobacterium Tuberculosis Complex* |
| 49 | Lumber spinal infection | *Haemophilus Parainfluenzae* |
| 51 | Salmonella blood infection | *Bena Collins* |
| 78 | Streptococcus pneumoniae bloodstream infection | *Moraxella non-liquefiable* |
| 84 | Mycobacterium tuberculosis bloodstream infection | *Bacteroides Vulgaris* |
| 89 | Mycobacterium tuberculosis bloodstream infection | *Bacteroides Fragilis* |

**S1B. False positive cases in NIID**

| **Case no** | **Final diagnosis** | **Pathogens detected by NGS** |
| --- | --- | --- |
| 98 | ANCA-related vasculitis | *Propionibacterium acnes* |
| 115 | Vasculitis | *Brucella* |
| 129 | Vasculitis | *Streptococcus mutans* |
| 125 | SLE | *Escherichia coli* |

**S1c. False positive cases in other kind of disease**

| **Case No** | **Final diagnosis** | **Pathogens detected by NGS** |
| --- | --- | --- |
| 153 | Crohn's disease | *Aspergilus Spp.* |
| 142 | Post infection allergy | *Fragile mycobacteria* |
| 146 | Fatigue syndrome | *Staphylococcus hominis* |
| 151 | Subacute thyroiditis | Mycobacterium Tuberculosis Complex |
